# Cross‐cultural variations in executive function impairments among children with attention deficit/hyperactivity disorder

**DOI:** 10.1002/jcv2.70032

**Published:** 2025-06-30

**Authors:** Da‐Wei Zhang, Ameera Shahira Amran, Yishu Qin, Han Jiang, Li Sun, Mark A. Bellgrove, Stuart J. Johnstone

**Affiliations:** ^1^ Department of Psychology Jeffrey Cheah School of Medicine and Health Sciences Monash University Malaysia Bandar Sunway Malaysia; ^2^ School of Education Yangzhou University Yangzhou China; ^3^ Suzhou Early Childhood Education College Suzhou China; ^4^ Peking University Sixth Hospital/Institute of Mental Health Beijing China; ^5^ National Clinical Research Center for Mental Disorders Key Laboratory of Mental Health Ministry of Health Peking University Beijing China; ^6^ School of Psychological Sciences and Turner Institute for Brain and Mental Health Monash University Melbourne Victoria Australia; ^7^ School of Psychology University of Wollongong Wollongong New South Wales Australia

**Keywords:** ADHD, cross‐cultural analysis, culture influence, executive function, latent profile analysis

## Abstract

**Background:**

Executive function (EF) impairments are heterogeneous in children with attention deficit/hyperactivity disorder (ADHD). Culture has a significant impact on EF development in typically developing (TD) children, yet its influence on EF impairments in those with ADHD remains understudied. This study aims to investigate the impact of cultural factors on EF impairments in children with ADHD through a cross‐cultural comparison.

**Methods:**

To ensure a robust sample size, the study initially recruited a large participant pool of 690 children from China and Australia. We applied similar diagnostic criteria and used propensity score matching to align clinical representation. This approach resulted in a final sample of 198 children aged 7–12, including 102 children diagnosed with ADHD and 96 TD peers. The same neuropsychological testing battery was used to assess EF in terms of working memory (WM), inhibitory control (IC), and set shifting.

**Results:**

Significant cultural effects were observed: Chinese children with ADHD showed lower performance in IC and WM compared to their TD peers, a pattern not seen in Australian children. A latent profile analysis revealed distinct EF profiles, highlighting a subgroup of Chinese children with severe EF impairments.

**Conclusions:**

This study advances cross‐cultural ADHD research on EF by using a robust methodology, including consistent diagnostic and testing procedures, propensity score matching, and person‐centered analysis. Our findings suggest that high‐EF‐expectation environments may have a negative effect on EF in children with ADHD, which provides insight into the underlying contributors to heterogeneous EF and underscores the need for culturally tailored ADHD interventions.

## Key points

**What's Known**

- Children with Attention Deficit/Hyperactivity Disorder (ADHD) often exhibit deficits in executive functions (EF); Meanwhile, EF deficits are not uniformly present in all children with ADHD, indicating heterogeneity within the disorder.
- Culture plays a significant role in the development of EF in typically developing (TD) populations.

**What's New**

- This study examines the impact of culture on EF deficits in children with ADHD, comparing Chinese and Australian populations, with rigorous methodologies, including propensity score matching and consistent diagnostic and neuropsychological testing procedures, to control for confounding variables and ensure reliable comparisons.
- The group comparison showed that Chinese children with ADHD showed lower performance in inhibitory control and working memory compared to their TD peers, a pattern not seen in Australian children. Distinct EF Profiles Identified:
- A latent profile analysis revealed distinct EF profiles, highlighting a subgroup of Chinese children with severe EF deficits.
- The study provides a nuanced understanding of how high‐EF‐expectation environments in Eastern cultures might exacerbate EF deficits in ADHD.

**What's relevant**

- The study highlights the role of culture in understanding EF presentation within the disorder.
- This study paves the way for further research into specific cultural dimensions that influence EF deficits, encouraging a more dimensional approach to understanding cultural impacts on ADHD.
- The findings highlight the need for ADHD interventions that consider cultural contexts, as the effectiveness of EF‐based interventions may vary across different cultural environments.

## Graphical abstract

This study explores the role of culture in executive function (EF) deficits in children with ADHD by comparing Chinese and Australian populations. Using rigorous methodologies such as propensity score matching and consistent diagnostic procedures, the research reveals distinct EF profiles, including a subgroup of Chinese children with severe EF deficits, particularly in inhibitory control and working memory. These findings underscore the influence of high‐EF‐expectation environments in Eastern cultures and suggest that ADHD interventions should account for cultural contexts to improve effectiveness.

## INTRODUCTION

Attention Deficit/Hyperactivity Disorder (ADHD) is a prevalent neurodevelopmental disorder characterized by impulsivity, hyperactivity, and/or inattention (APA, 2013). ADHD often results in reduced quality of life (Schwörer et al., 2020) and poor academic performance (Tamm et al., 2021). Several causal models attribute ADHD symptomatology to impairments in executive functions (EF), the cognitive abilities needed to regulate behavior (Barkley, 1997; Sergeant, 2005; Sonuga‐Barke, 2005). Notably, not all children with ADHD show EF impairments, and those who exhibit differing patterns indicate EF heterogeneity within the condition (Nigg et al., 2020). There is, however, no clear explanation for these heterogeneous EF impairments in ADHD.

Recent research increasingly points to the role of culture in shaping EF. Culture permeates every aspect of human development, providing a framework within which individuals learn to prioritize and regulate behavior and cognition (Kitayama & Uskul, 2011). Cognitive abilities, including EF, are significantly shaped by cultural evolution through social learning and interaction (Heyes, 2018). The development of EF is thought to occur due to values perceived and recognized by an individual's sociocultural environment (Chiao, 2018; Kitayama & Uskul, 2011). Children from Eastern societies, which generally prioritize group harmony and discipline, perform better in EF tasks requiring a high level of inhibitory control (IC) than their Western counterparts, who are typically raised in environments that value individualism and independence (Ellefson et al., 2017; Fujita et al., 2022; Grabell et al., 2015; Schmitt et al., 2019; Tran et al., 2019). The difference can be attributed to the higher expectations associated with Eastern societies, which emphasize IC in societal practices, including peer relations, parent‐child interactions, and educational systems, leading to a collective improvement in IC at the individual level (Kitayama & Uskul, 2011; Qu et al., 2021). It has become increasingly apparent that culture plays an important role in EF development and that integrating cultural perspectives could improve theoretical models of the development of EF (Doebel, 2020).

Considering the influence of cultural values on typical populations, it is plausible to assume that cultural variations could also contribute to the manifestation of EF impairments in children with ADHD. Although ADHD core symptoms are consistent across cultures (Bauermeister et al., 2010), cultural differences may influence their expression (APA, 2013; Faraone et al., 2021). It appears, despite limited studies, that culture may play a moderating role in EF impairments among ADHD. Cross‐cultural comparisons have revealed significant differences in EF among children with ADHD from various countries, suggesting cultural impacts on these cognitive processes (Alloway & Cockcroft, 2014; Glozman & Shevchenko, 2014; Thorell et al., 2013). However, several methodological issues challenge the findings: (1) ADHD severity and presentation, as well as a diverse demographic background, may confound results; (2) the absence of control groups in some studies raises questions regarding whether these effects are specific to ADHD; and (3) the limited or atypical selection of EF tasks used in studies necessitates a more detailed examination of how culture impacts ADHD manifestations.

The current research addresses methodological issues by conducting a cross‐cultural study between typically Eastern and Western contexts, using samples from China and Australia, to examine the influence of cultural factors on EF impairments in children with ADHD. A similar diagnostic procedure is applied across cultural contexts for a reliable comparison. A propensity score matching approach equates groups based on ADHD severity and presentation types. This approach helps to mitigate confounding variables related to variations in ADHD symptomatology and severity across cultural groups. The same neuropsychological battery measures EF—specifically inhibition, working memory (WM), and switching—across all participants. This standardized approach ensures that any observed differences in EF can be attributed more confidently to cultural influences rather than discrepancies in the assessment. Furthermore, children with ADHD are compared to their local counterparts without ADHD, allowing for a direct investigation of how cultural contexts influence EF within the same geographical area.

In addition to the conventional group‐level comparison, this study employs a person‐centered approach to investigate how culture shapes EF profiles in ADHD. Person‐centered analysis focuses on identifying subgroups within a population based on shared characteristics, rather than assuming that all individuals within a group exhibit the same performance pattern (Woo et al., 2024). This approach is particularly relevant given the well‐documented heterogeneity of EF impairments in ADHD (Nigg et al., 2020). Empirical studies have identified multiple distinct EF profiles within ADHD populations, although the specific profiles vary depending on the EF measures used (e.g., Fair et al., 2012; Roberts et al., 2017; Rosello et al., 2020; Vaidya et al., 2020; van Hulst et al., 2015). These EF profiles have been shown to be more sensitive in detecting cognitive impairments than the ADHD diagnostic category (Nigg et al., 2020; Roberts et al., 2017; Vaidya et al., 2020). Notably, a behavioral genetics study suggests that environment plays a crucial role in shaping EF profiles in children with ADHD (Arnett et al., 2022). Thus, the present study further examines whether culture moderates EF profiles in ADHD. Together, the complementary use of person‐centered analysis alongside conventional group‐level cross‐cultural comparisons offers a more comprehensive understanding of the interactions between culture and EF in children with ADHD.

Substantial research indicates that children from Eastern cultures often demonstrate stronger EF than their Western counterparts, likely due to societal values emphasizing perseverance and delayed gratification (e.g., Theule et al., 2013; Zhang et al., 2021). Consequently, we hypothesize that Chinese children with ADHD will exhibit smaller EF impairments relative to Australian children with ADHD, a difference we expect to emerge consistently across both the conventional group‐level comparison and the person‐centered analysis.

## METHODS

### Participants

This study used baseline data from two larger projects conducted at the University of Wollongong in collaboration with Peking University and Zhejiang Normal University. Both children with and without ADHD included in the study had IQ scores above 80 and ranged in age from 7 to 12 years. General exclusion criteria were: (a) a diagnosis or history of head trauma with loss of consciousness, (b) a history of neurological illness or other severe disease, and (c) a diagnosis of schizophrenia, affective disorders, anxiety, tic disorders, pervasive developmental disorders, or intellectual disability. For the ADHD group, all participants were recently diagnosed and had not yet undergone any medication treatment. For the control group, only children without AD/HD or any other psychological or psychiatric diagnosis were included, as indicated via a semi‐structured interview with the child's parent(s). This was further verified using the SNAP‐IV, with children showing elevated ADHD symptoms excluded. Ethical approval for this research was granted by the Peking University Sixth Hospital Ethics Committee, Zhejiang Normal University Ethics Committee, the Human Research and Ethics Committee at the University of Wollongong (HE, 2018/400), and the Monash University Human Research Ethics Committee (ID: 38457).

Children with ADHD in China were recruited from the Peking University Sixth Hospital in Beijing. A child and adolescent psychiatrist conducted the initial clinical diagnosis. Subsequent validation used the Chinese version of the Schedule for Affective Disorders and Schizophrenia for School‐Age Children Present and Lifetime Version (K‐SADS‐PL; Kaufman et al., 1997), applying criteria from the Diagnostic and Statistical Manual of Mental Disorders (APA, 2013). Two senior child and adolescent psychiatrists independently completed the diagnosis, achieving high interrater reliability with kappa values ranging from 0.80 to 1.00.

Children with ADHD in Australia were recruited from the Wollongong and southern Sydney area through advertisements placed in the offices of General Practitioners and Psychologists, as well as on community noticeboards and various social media groups. These advertisements sought the participation of children both with and without a professional diagnosis of ADHD. For children with ADHD, diagnoses were established by clinical professionals (i.e., psychologists, psychiatrists, or pediatricians) through comprehensive evaluations. These assessments considered behavioral symptoms and functional impairment using multi‐informant ratings from parents and teachers, along with clinical interviews and observations. Diagnoses were made in accordance with DSM‐5 criteria.

One key manipulation in this study was to ensure that children with ADHD from the two countries were comparable in terms of symptoms. Children with ADHD were matched based on ADHD symptom severity (measured by the Conners' Index scores introduced below) and presentation type (i.e., predominantly hyperactive/impulsive, predominantly inattentive, or combined). The matching was done using optimal matching, a propensity score method to balance observed covariates between the groups (Rosenbaum, 1989). This approach ensures that any observed differences in EF performance can be more accurately attributed to cultural influences rather than differences in ADHD presentation or severity.

The control groups consisted of children drawn from local public schools in each country. Consent for school participation was obtained from both headmasters and parents' committees of the involved schools. Study details were disseminated to parents interested in their children's potential participation. Non‐clinical control children met the following criteria: no diagnosis of ADHD or other neurodevelopmental disorders and completed all neuropsychological tests administered to children with ADHD.

**FIGURE 1:**
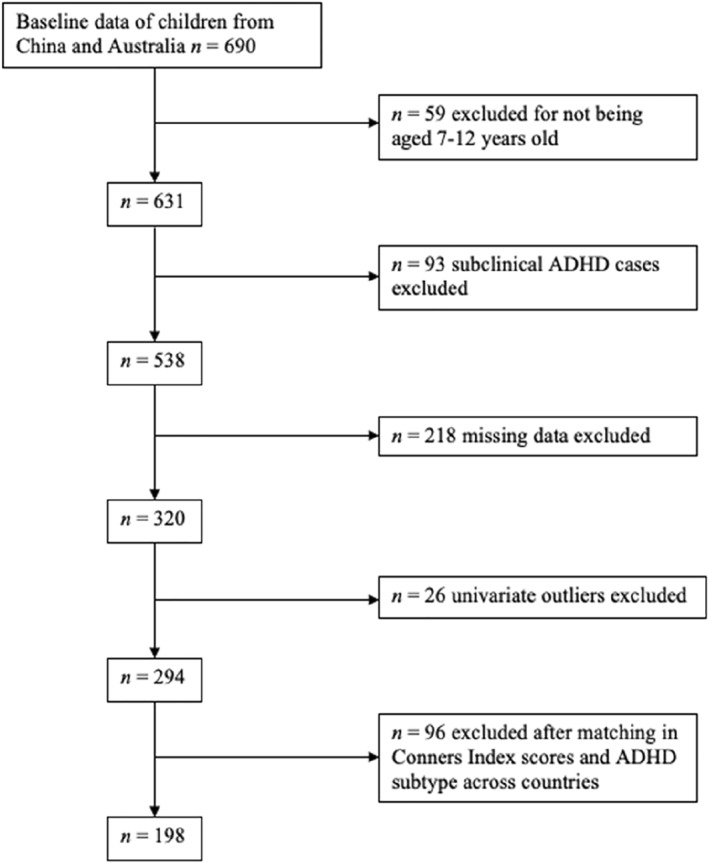
Flowchart of data screening.

The achieved data pool initially involved 690 children. Figure 1 shows the data‐screening steps we followed. Briefly, after excluding participants outside the specified age range, removing controls whose parents reported elevated ADHD symptoms (as detailed in the ADHD diagnosis section), addressing missing data, excluding univariate outliers on neuropsychological tests, and matching the propensity score based on Conners' Index scores and ADHD subtype, a final sample of 198 children was obtained (Please see Table 1 for details)S. Further statistical details are provided in the Statistical Analysis section. The total sample size of 198 children met the requirements identified by a G*Power v3.1 analysis for conducting a two‐way analysis of variance (ANOVA), with parameters set at *α* = 0.05, a medium effect size (*ƒ* ^*2*^ = 0.24), and a power of 0.80. This sample size was determined to be sufficient to detect the hypothesized effects, informed by previous cross‐cultural research on ADHD and EF (Chan et al., 2022; Ellefson et al., 2017; Marsh et al., 2015).

**TABLE 1:** Means, standard deviations, frequencies, and proportions of participant demographic characteristics.

	China		Australia	
	TD	ADHD	TD	ADHD
	(*n* = 51)	(*n* = 48)	(*n* = 51)	(*n* = 48)
Age (years), *M* (SD)	9.1 (1.73)	9.12 (1.44)	8.78 (1.53)	9.29 (1.46)
Gender, *n* (%)				
Male	27 (53)	39 (81)	25 (49)	30 (62)
Female	24 (47)	9 (19)	26 (51)	18 (38)
ADHD subtype, *n* (%)				
Combined	–	20 (42)	–	20 (42)
Predominantly inattentive	–	22 (46)	–	22 (46)
Predominantly hyperactive/Impulsive	–	6 (12)	–	6 (12)
Conners index score, *M*(SD)	7.06 (3.91)	16 (4.69)	7 (4.62)	16.02 (4.80)

*Note*: *N* = 198.

Abbreviations: ADHD, attention deficit hyperactivity disorder; *M*, mean; SD, standard deviation; TD, typically developing.

### Materials

#### ADHD diagnosis and symptom severity

The diagnostic framework for ADHD involved the Swanson, Nolan, and Pelham (SNAP‐IV) rating scale (Swanson, 1992) alongside a clinical diagnosis according to DSM‐5 criteria (APA, 2013). For children with ADHD, diagnoses were established during routine clinical assessments in each country. For control children, ADHD was ruled out via parent report, which was verified by examining parent ratings on each of the 18 diagnostic criteria within the SNAP‐IV. We verified the diagnosis provided by the parent (usually in the form of a written report from a psychologist, psychiatrist, or pediatrician) by reviewing ratings on all 18 criteria. Ratings of “very much” (on a scale ranging from “Not at all” to “Very much”) were considered indicative of a criterion being met. Participants meeting six or more criteria in both the Inattentive and Hyperactive/Impulsive domains were classified as having ADHD‐C; those meeting six or more criteria only in the Inattentive domain were classified as ADHD‐I; and those meeting six or more criteria only in the Hyperactive/Impulsive domain were classified as ADHD‐HI.

The Conners' Index Subscale was also employed to assess ADHD symptom severity. Parents rated the items on a 4‐point Likert scale spanning from 0 (“Not at all”) to 3 (“Very much”). Scores were totaled to a final score out of 30, with higher scores representing greater ADHD symptom severity. The parent form of the Conners' Index Scale demonstrates high internal consistency (*α* = 0.91) and convergent validity in school‐aged Chinese children with ADHD (Gau et al., 2006) and is robust against cultural influences (Schmidt et al., 2017).

#### Computerized EF tasks

We developed and employed an in‐house computerized platform to assess EF based on current understanding of EF structure. This platform specifically measures WM, IC, and set shifting (SS) using established task paradigms. To better capture cognitive performance in children, the platform integrates child‐friendly gamification features to enhance engagement and adaptive progression systems that dynamically adjust task difficulty, minimizing floor and ceiling effects. Additionally, the platform features bilingual interfaces in Chinese and English. To ensure cross‐cultural comparability, task instructions were rigorously translated and back‐translated by two bilingual psychological researchers fluent in both languages. Earlier validation work demonstrated that the platform can distinguish children with ADHD from TD controls in a Chinese sample (Johnstone et al., 2021). Outcome variables in the current study were selected based on this previous validation.

WM was examined with a Search task derived from Morris et al. (1988). Children were instructed to select virtual doors (2‐6 doors) until a person was found behind one. Each time a person was found, they were re‐hidden behind another door, and the search resumed until all doors of that block had been used. Search rules required the children not to select the same door more than once or doors behind which a person had been found. The task involved three blocks of varying difficulties; block 1 began with 4 doors (Level 3). If an error occurred, the following block proceeded at a lower level: 3 doors (Level 2) or 2 doors (Level 1). If no errors occurred, the following block proceeded at a higher level: 5 doors (Level 4) or 6 doors (Level 5). WM performance score was quantified by summing the levels successfully completed across the three adaptive blocks, with higher scores indicating better performance.

IC was examined using the Go/No‐go task derived from Benikos et al. (2013). Children were instructed to click the mouse button during “Go” trials (food images) and refrain from clicking during “No‐go” trials (non‐food images). Since Go trials constituted 80% of trials, a prepotent response tendency was created; children were to inhibit this response during No‐go trials. The task involved three blocks equally distributed with 42 stimuli. The order of presented stimuli was pseudo‐random, that is, no blocks started with a No‐go trial, and no more than two consecutive No‐go trials occurred. In block 1, the stimulus presentation lasted 800 ms (Level 3). If an error occurred, the following block proceeded at a lower level: 1200 ms (Level 2) or 1600 ms (Level 1). If no errors occurred, the following block proceeded at a higher level: 600 ms (Level 4) or 400 ms (Level 5). IC performance scores were determined by summing levels completed across the three blocks, with higher scores indicating superior IC performance.

SS was examined using an object sorting task derived from Howard and Melhuish (2017). Children were instructed to sort objects (blue square, red square, blue circle, red circle) by a sorting rule (shape or color). The task involved three blocks, each comprising 15 trials. No color‐shape combination was repeated for more than two consecutive occurrences for each block. In block 1, children sorted objects according to color, that is, blue or red. In block 2, children sorted objects according to shape, that is, square or circle. Block 3 required children to switch rules according to the object's border flexibly; a black‐bordered object was sorted by shape, while a non‐black‐bordered object was sorted by color. SS performance was measured by flexible switch reaction time, that is, average time in milliseconds for correct sorting of block 3 objects. A shorter reaction time indicated better performance.

### Procedure

Recruitment was conducted via convenience sampling, with advertisements in local hospitals, general practitioners' offices, and primary schools within each country. In China, children with ADHD were recruited from Peking University Sixth Hospital and TD children from local schools in Hangzhou and Zhejiang province. In Australia, children were recruited from clinics, hospitals and schools in Wollongong and southern Sydney. After obtaining consent from parents through a detailed participant information sheet, the children were assessed individually in a controlled setting, typically at their local school or a hospital. Each session lasted approximately 30–35 min, during which the children engaged in the EF tasks under the supervision of a trained research assistant.

### Statistical analysis

Data analysis was conducted using R and Mplus. Missing data were handled using listwise deletion, as all missing values concerned the parent‐rated Conners Index—a critical variable for matching participants on ADHD severity—and Little's MCAR test confirmed that data were missing completely at random; importantly, the remaining sample met the sample size estimate. Univariate outliers were identified and removed from each outcome measure via boxplot inspection. Based on propensity scores (Rosenbaum, 1989), optimal matching was employed to ensure that children with ADHD from both cultural settings were comparable in terms of Conners' Index scores and subtype. Preliminary analyses of demographic characteristics were conducted to identify any significant differences between comparison groups. Independent sample *t*‐tests analyzed continuous variables, while chi‐square tests analyzed categorical variables.

The primary statistical method was a 2 × 2 factorial ANOVA to explore interactions between Country and ADHD diagnosis across the three EF measures. The normality of data was assessed using Shapiro‐Wilk tests, and the homogeneity of variance was checked via Levene's tests. Where necessary, log transformations were applied to meet the assumptions of ANOVA. The significance of ANOVA findings was interpreted using effect sizes η_*p*_^2^. The effect size values were interpreted as small (0.01), medium (0.06), or large (0.14). Post‐hoc simple effects analyses were conducted to determine the direction of any significant results, using the Bonferroni correction to control for familywise error rate and Cohen's *d* to estimate effect size. Cohen's *d* values were interpreted as small (0.2), medium (0.5), or large (0.8). All other statistical analyses were conducted with a significance level set at *α* = 0.05.

To complement the group‐level analysis, a person‐centered analysis was conducted to identify potential EF cognitive subtypes within the sample. While several methods exist for identifying such profiles, a recent simulation study by Agelink van Rentergem et al. (2022) demonstrated that Latent Profile Analysis (LPA) remains particularly robust in scenarios with sample sizes ranging from 100 to 200 and a limited number of indicators—conditions that closely mirror our study (*n* = 198 with 3 indicators). Thus, LPA was employed here. Each EF task performance was Z‐transformed based on the means and standard deviations of the TD children. This standardization ensures that all LPA outcomes are interpreted relative to the performance of TD children. The standardized scores of the Z‐transformed EF performance were used as the indicators for the latent profile membership. Models with 2–5 profiles were computed to identify the optimal number of subgroups. Several criteria were used to determine the best‐fitting model, including the Akaike Information Criterion (AIC), Bayesian Information Criterion (BIC), and Sample‐size adjusted BIC (SaBIC), with lower values indicating better fit (Nylund et al., 2007). The optimal number of profiles was determined based on guidance from several criteria (Nylund et al., 2007). Additionally, the Lo‐Menddell‐Rubin test (LMRT) and bootstrapped likelihood ratio test (BLRT) were conducted for each solution to compare the *k*−1 versus *k* class model, with a non‐significant LMRT and BLRT test supporting a model with one less profile. The entropy value (ranging from 0 to 1) was used to indicate classification accuracy, with higher values representing greater precision in classification. It is worth noting that simulation studies have shown that some statistical indices (i.e., BIC, ABIC, BLRT) are far more effective than others and should be prioritized in selecting the optimal number of profiles (Marsh et al., 2009). The theoretical interpretability of the profiles was also considered. Once the optimal number of profiles was determined, multinomial logistic regressions were conducted to examine the effects of ADHD diagnosis, Country, and their interaction on profile membership.

## RESULTS

### Preliminary analyses

Independent‐samples *t*‐tests were used to determine any significant differences in age and Conners' Index scores across comparison groups. Children from China and Australia did not differ in age and Conners' Index scores regardless of ADHD diagnosis (*p* > 0.05). Within both countries, children with ADHD and TD children did not differ in age (*p* > 0.05). There were significant differences in Conners' Index scores between children with ADHD and TD children in China (*t* (92) = 10.28, *p* < 0.001, Cohen's *d* = 2.07) and Australia (*t* (96) = 9.51, *p* < 0.001, Cohen's *d* = 1.91), constituting large effects. Chi‐square comparisons were used to determine significant differences in ADHD subtype and gender across comparison groups. Children with ADHD from China and Australia did not differ in ADHD subtype (*p* > 0.05). Significant differences were found in gender in children with ADHD across countries, χ^2^ (1, *N* = 96) = 4.17, *p* < 0.05. That is, there was a larger proportion of males with ADHD in the Chinese cohort, χ^2^ (1, *N* = 99) = 8.92, *p* < 0.01. Despite these differences, the literature presents mixed evidence regarding the influence of gender on EF outcomes in ADHD (Bálint et al., 2009; Biederman et al., 2005; Loyer Carbonneau et al., 2021). We examined its influence by including gender as a covariate in our analyses for each EF measure and found no significant main effect or interaction for gender. Thus, we proceeded with models that did not control for sex. Descriptive statistics of EF task performance by comparison groups are presented in Table 2.

**TABLE 2:** Means and standard deviations for working memory, inhibitory control and set shifting.

	China		Australia	
	TD	ADHD	TD	ADHD
	(*n* = 51)	(*n* = 48)	(*n* = 51)	(*n* = 48)
Working memory, *M* (SD)	10.59 (1.51)	9.54 (2.23)	10.12 (1.62)	10.27 (2.07)
Inhibitory control *M* (SD)	11.14 (1.46)	9.21 (2.32)	10.63 (1.85)	10.40 (1.94)
Set shifting, *M* (SD)	8.05 (0.36)	8.09 (0.37)	8.03 (0.30)	7.99 (0.29)

*Note*: *N* = 198; working memory and inhibitory control scores represent performance scores; set shifting scores represent log‐transformed reaction time.

Abbreviations: ADHD, attention deficit hyperactivity disorder; *M*, mean; SD, standard deviation; TD, typically developing.

### Group comparisons

Separate 2 × 2 factorial ANOVAs were conducted to examine the effects of Country and ADHD diagnosis on IC, WM, and SS.

#### Inhibitory control

**FIGURE 2:**
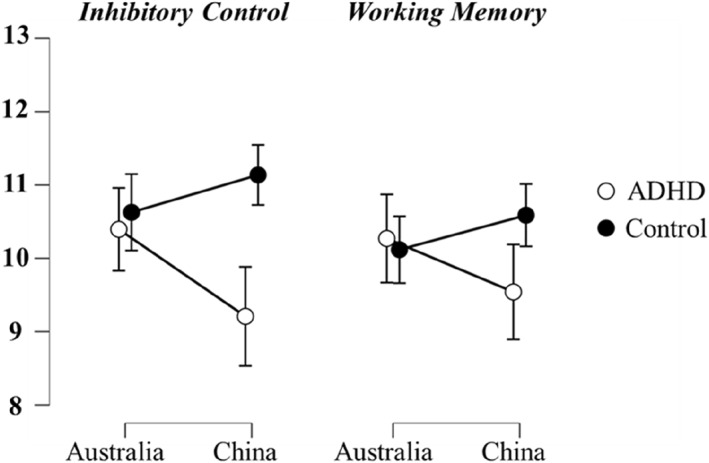
Interaction plots between attention deficit/hyperactivity disorder diagnosis and Country for inhibitory control and working memory. Bars indicate 95% confidence interval. *Y*‐axis represents the performance score.

No significant main effect was found for Country, *F* (1, 194) = 1.33, *p* = 0.251, *η*_*p*_^2^ = 0.01. There was a significant main effect for ADHD diagnosis, *F* (1, 194) = 15.78, *p* < 0.001, *η*_*p*_^2^ = 0.08. A statistically significant interaction indicated that the effects of ADHD diagnosis on IC performance scores depended on Country, *F* (1, 194) = 9.74, *p* = 0.002, *η*_*p*_^2^ = 0.05. The effect is illustrated in Figure 2. Post hoc simple effects analyses using a Bonferroni correction showed that IC performance scores were significantly worse in Chinese children with ADHD than in Chinese TD children, *t* (78) = −4.91, *p* < 0.001, Cohen's *d* = 1.00. Meanwhile, Australian children with ADHD and TD children did not differ in IC performance scores, *t* (96) = −0.61, *p* = 0.546, Cohen's *d* = 0.12.

#### Working memory

No significant main effect was found for Country, *F* (1,194) = 2.81, *p* = 0.677, *η*_*p*_^2^ < 0.001, nor ADHD diagnosis, *F* (1,194) = 0.17, *p* = 0.095, *η*_*p*_^2^ = 0.01. A statistically significant interaction indicated that the effects of ADHD diagnosis on WM performance scores depended on Country, *F* (1,194) = 5.07, *p* = 0.026, *η*_*p*_^2^ = 0.03. The effect is presented in Figure 2. Post hoc simple effects analyses using a Bonferroni correction indicated that WM performance scores were significantly worse in Chinese children with ADHD than in Chinese TD children, *t* (82) = −2.72, *p* = 0.008, Cohen's *d* = 0.55. In contrast, Australian children with ADHD and TD children did not differ in WM performance scores, *t* (89) = 0.41, *p* = 0.684, Cohen's *d* = 0.08.

#### Set shifting

No significant main effect was found for Country, *F* (1,194) = 1.82, *p* = 0.179, *η*_*p*_^2^ = 0.01, nor for ADHD diagnosis, *F* (1,194) = 0.00, *p* = 0.963, *η*_*p*_^2^ < 0.001. Likewise, the interaction between Country and ADHD diagnosis on SS reaction time was non‐significant, *F* (1,194) = 0.70, *p* = 0.405, *η*_*p*_^2^ < 0.001.

### Person‐centered analysis

**FIGURE 3:**
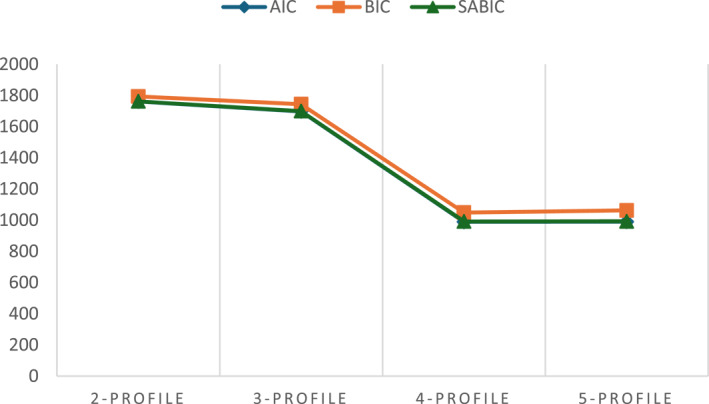
Elbow plot of Akaike information criterion, BIC, and sample‐size adjusted BIC values for 2–5 profile solutions. Information criteria tend to decrease as additional classes are extracted (Nylund et al., 2007). The elbow plot visualizes the point at which the improvement in fit begins to plateau—here at the four‐profile solution—indicating that further profiles confer minimal gains (Sinha et al., 2021). BIC, Bayesian information criterion.

The four‐profile model was selected as the optimal solution as it achieved the lowest AIC, BIC, and SABIC values among the 2–5 class models and the highest entropy (Table 3), while both BLRT and LMR tests became non‐significant for the jump from four to five profiles, indicating no further fit improvement. Consistent with these indices, an elbow plot of AIC, BIC, and SABIC against profile number revealed a clear inflection at four classes, marking the point of diminishing returns (Figure 3). Moreover, two profiles (*n* = 40 and *n* = 105) exhibited identical parameter estimates when comparing three‐ and four‐class solutions, further underscoring the stability and robustness of the selected model.

**TABLE 3:** Model fit indices for the latent profile classification with 2–5 classes.

Profile	AIC	BIC	SABIC	pLMR	pBLRT	Entropy	Group size
2	1761.23	1794.11	1762.43	0.00	0.00	0.95	53/145
3	1698.04	1744.08	1699.73	0.18	0.00	0.99	53/40/105
**4**	**989.79**	**1048.98**	**991.96**	**0.77**	**0.00**	**1.00**	**16/37/40/105**
5	990.67	1063.01	993.32	0.32	0.38	0.98	31/16/105/40/6

*Note*: Values in bold indicate the selected model.

Abbreviations: AIC, Akaike information criterion; BIC, Bayesian information criteria; BLRT, Bootstrapped likelihood ratio test; LMR, Lo‐Menddell‐Rubin test; SaBIC, Sample‐size adjusted bayesian information criteria.

**FIGURE 4:**
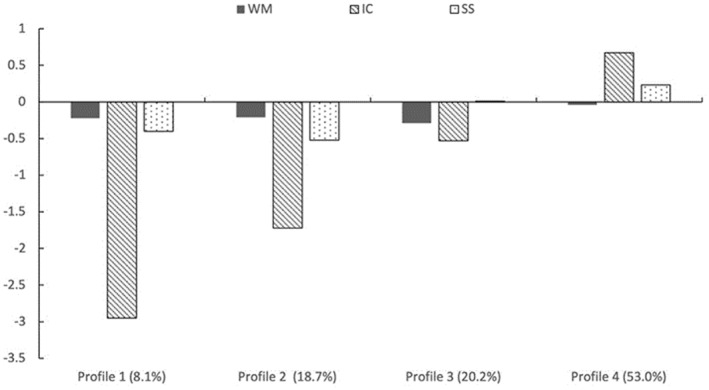
Characteristics of the executive functioning profiles. IC, inhibition control; SS, set shifting; WM, working memory.

The four identified EF profiles (Figure 4) were as follows: Profile 1—the severe low EF group (11 Chinese children with ADHD, 3 Australian children with ADHD, and 2 Australian typically‐developing children) represented 8.1% of the total sample and exhibited extremely low IC at about −3 SD, and mild low WM and SS at about −0.5 SD. Profile 2—the moderate low EF group (12 Chinese children with ADHD, 7 Chinese typically‐developing children, 8 Australian children with ADHD, and 10 Australian typically‐developing children) accounted for 18.7% of participants, characterized by moderate low IC at about −1.5 SD, and mild low WM and SS at about −0.5 SD. Profile 3—the mild low EF group (10 Chinese children with ADHD, 8 Chinese typically‐developing children, 13 Australian children with ADHD, and 9 Australian typically‐developing children) included 20.2% of the total sample and was characterized by mild low WM and SS, both at about −0.5 SD. Profile 4—the normative EF group (15 Chinese children with ADHD, 36 Chinese typically‐developing children, 24 Australian children with ADHD, and 30 Australian typically‐developing children) comprised 53.0% of the sample and scored highest on all three EF tasks, with performance within 0.5 SD of the mean.

A multinomial logistic regression analysis was conducted to predict LPA generated profiles based on ADHD diagnosis, Country, and their interaction. Like group level comparisons, this study focused on the interaction between ADHD diagnosis and Country. There was a significant interaction χ^2^ (9, *N* = 198) = 30.94, *p* < 0.001, indicating that the relationship between ADHD diagnosis and profiles differs between China and Australia.

Profile 4 was set as the reference category, given that it was characterized by typical EF. The log odds of each other profile were compared to it of the profile 4. The interaction term was only significant for predicting Profile 1, *B* = 1.769, SE = 0.730, Wald χ^2^ (1) = 5.878, *p* = 0.015, suggesting that children with ADHD in China had significantly higher odds (Exp(*B*) = 5.867, 95% CI [1.403, 24.523]) of being in the profile 1, the one characterized by extremely low IC and mild low WM and SS.

## DISCUSSION

This study aimed to investigate the influence of culture on executive function (EF) impairments in children with ADHD by conducting a cross‐cultural comparison between children from China and Australia. We found that Chinese children with ADHD exhibited significantly lower IC and WM performance than their TD peers. In contrast, Australian children did not demonstrate any differences. Further, the person‐centered analysis revealed a specific low EF performance group in Chinese children with ADHD.

Methodological improvements have led this study to highlight the role that cultural factors play in modulating EF impairments in children with ADHD. While the influence of culture on cognitive development has been well‐documented (Chiao, 2018; Doebel, 2020; Kitayama & Uskul, 2011), few studies have examined the influence of culture on EF impairments in children with ADHD, often with methodological limitations. Broadly speaking, our results align with earlier findings (Alloway & Cockcroft, 2014; Glozman & Shevchenko, 2014), suggesting that culture moderates EF impairments in ADHD. This argument is further supported with methodological improvements in this study. The current study defined “impairments” by comparing ADHD participants to their local counterparts, thus avoiding the complications of directly comparing ADHD across different countries where differences may be attributed to general cultural or environmental influences on EF rather than ADHD‐specific factors. Moreover, the severity and presentation of ADHD were well controlled using similar recruitment approaches and propensity score matching, thereby ruling out the possibility that differences in diagnosis caused the observed variations. Furthermore, using the same battery to measure EF impairments systematically, our results indicate that culture particularly influences IC and WM impairments.

The LPA results provided additional insight by identifying unique clusters within the sample to complement the group‐level comparisons. Compared to group‐level comparison (i.e., a variable‐centered approach) which focuses on average group differences, LPA (i.e., a person‐centered approach) provides a more nuanced understanding of individual differences, ensuring that nuanced patterns are not overlooked (Muthén & Muthén, 2000). We identified a distinct subgroup of Chinese children with ADHD, characterized by low IC and mild low WM and SS, compared to their Australian and Chinese peers who are TD. Thus, both group‐level and person‐centered analyses implied the same conclusion: cultural factors moderate the effects of ADHD on EF. This finding aligns with previous behavioral genetics research suggesting that EF profiles in ADHD are not stable phenotypic traits (Arnett et al., 2022). Our results further indicate the role of culture in profiling EF in children with ADHD, highlighting the need for future research to adopt a culture‐sensitive approach in disentangling environmental contributions to EF variability in ADHD.

Our results suggest that a high‐EF‐expectation culture may have negative impacts on EF in children with ADHD. Substantial research indicates that children from Eastern cultures typically have better EF due to societal values that encourage behaviors supporting EF development, such as perseverance and delayed gratification (Theule et al., 2013; Zhang et al., 2021). One might expect these values to mitigate EF impairments in children with ADHD. However, we observed the opposite: significant EF impairments in Chinese children with ADHD. This finding suggests that a high‐EF‐expectation environment might exacerbate EF impairments in ADHD. From a developmental perspective, environmental factors (e.g., parenting) and experiences (e.g., opportunities to exercise EF) are crucial in shaping EF development, as highlighted by theories like interactive specialization (Johnson, 2011) and skill learning (Klingberg, 2014). The dynamic developmental perspective of ADHD suggests that the nature of ADHD, such as aversion to delays essential in EF tasks, places children at a disadvantage (e.g. Sagvolden et al., 2005; Sonuga‐Barke, 2005). As a result, they avoid situations that require EF, resulting in fewer opportunities for EF practice and negative feedback from situations requiring EF. From this dynamic developmental perspective, we suspect that when high EF expectations exist, reduced opportunities and increased negative feedback may further hinder EF development in ADHD. As an example, parents of children with ADHD in Hong Kong experience a higher level of parental stress than those in the UK (Chan et al., 2022). This stress has been shown to affect parent‐child interaction and development of EF in children with ADHD (Valcan et al., 2018). Likewise, teacher‐student interactions, which have a significant impact on EF development (Vandenbroucke et al., 2018), are less favorable in South Korea for children with ADHD than in Germany (Lee & Witruk, 2016). Please note that our study focused on laboratory‐based EF measures (Salehinejad et al., 2021). Future research could extend these findings by assessing EF in daily life, which may capture different aspects of EF (Bakar et al., 2011), thus offering a deeper understanding of how culture moderates EF.

The absence of significant differences in EF impairments between ADHD and TD children in Australia does not imply that EF impairments are unimportant for ADHD in Western cultures. Numerous neuropsychological and neuroimaging studies report EF impairments in Western populations with ADHD (Faraone et al., 2024). These results may, however, have been influenced by the selection of outcomes derived from our neuropsychological testing battery. This study recruited participants from a wide age range and used a more holistic outcome measure—difficulty level, indexing the highest challenge level a participant could complete. While this choice avoids range restrictions across different age groups, it may lose sensitivity to more nuanced behavioral observations like reaction time or computational modeling‐based measures.

A further limitation of this study is the complexity of cross‐cultural comparisons. Culture is much more nuanced than the simple Eastern versus Western dichotomy. The study did not incorporate measures that reflect cultural dimensions, so we cannot draw conclusions regarding which specific cultural factors influenced the observed differences. A dimensional approach is emerging in cultural psychology to determine the exact cultural dimensions that influence cognition (Kitayama & Salvador, 2024). Future studies should measure cultural dimensions, such as the tightness versus looseness of social norms, to examine the mechanisms underlying cross‐cultural differences. Also, it should be noted that both Australia and China are culturally diverse; the current study does not account for cultural variations within each country.

Another limitation involves the analytic approach used to examine associations between latent profiles and external variables. Ideally, such associations should be assessed with methods explicitly accounting for classification uncertainty (Nylund‐Gibson et al., 2019). However, uneven participant distributions across latent profiles and external categories (e.g., no TD Chinese children in Profile 1) led to estimation non‐identifiability issues. Thus, we employed a three‐step analytic approach (LPA followed by ANOVA), which, while not fully addressing classification uncertainty, yielded robust and interpretable findings appropriate to our data. Previous literature supports the use of this three‐step procedure as an alternative under similar conditions (Asparouhov & Muthén, 2014; Nylund‐Gibson et al., 2019). Future studies could verify our findings in a sample enabling analytic methods that fully address classification uncertainty.

Despite these limitations, the rigorous methodology of this study demonstrates the influence of culture on EF impairments in ADHD, offering several implications. To the best of our knowledge, it is the first study to demonstrate that culture moderates EF impairments in ADHD through a rigorous cross‐cultural comparison. It is widely accepted that not all children with ADHD suffer from EF impairments (Nigg et al., 2020), but its causes are little known. We identify culture as one such source that requires further study, in terms of specific dimensions and pathways through which culture moderates EF impairments. Furthermore, targeting EF has been a key strategy for developing alternative interventions for ADHD (Zhang, 2023; Zhang et al., 2023). The results of this study suggest that EF‐based interventions should consider cultural background since such interventions may be particularly effective in certain cultural contexts but not in others.

This study aimed to examine the impact of cultural context on EF impairments in children with ADHD by comparing samples from China and Australia. Chinese children with ADHD displayed significantly lower IC and WM than their TD peers based on the group comparisons, a pattern not observed in Australian children. Besides, the person‐centered analysis identified a distinct EF impairment profile that was more prevalent among Chinese children with ADHD. These results suggest that high‐EF‐expectation cultures may exacerbate EF impairments in children with ADHD, highlighting the importance of factoring cultural context when considering EF impairments in ADHD. Our study highlights the need for culturally tailored approaches to better support children with ADHD in diverse cultural settings.

## AUTHOR CONTRIBUTIONS

**Da‐Wei Zhang:** Conceptualization; data curation; formal analysis; investigation; methodology; project administration; resources; software; supervision; validation; visualization; writing—original draft; writing—review and editing. **Ameera Shahira Amran:** Formal analysis; investigation; methodology; project administration; software; visualization; writing—original draft. **Yishu Qin:** Conceptualization; formal analysis; visualization; writing—original draft. **Han Jiang:** Conceptualization; methodology; resources; writing—original draft. **Li Sun:** Conceptualization; data curation; methodology; resources; writing—original draft. **Mark A. Bellgrove:** Conceptualization; methodology; resources; supervision; writing—original draft. **Stuart J. Johnstone:** Conceptualization; data curation; formal analysis; methodology; project administration; resources; software; writing—original draft; writing—review and editing.

## CONFLICT OF INTEREST STATEMENT

The authors declare no conflicts of interest.

## ETHICAL CONSIDERATIONS

Ethical approval for this research was granted by the Peking University Sixth Hospital Ethics Committee, Zhejiang Normal University Ethics Committee, the Human Research and Ethics Committee at the University of Wollongong (HE 2018/400), and the Monash University Human Research Ethics Committee (ID: 38457). Consent for school participation was obtained from both headmasters and parents' committees of the involved schools. Consent was obtained from parents through a detailed participant information sheet.

## Data Availability

The data that support the findings of this study are available from the corresponding author upon reasonable request.
